# Effectiveness of auriculotherapy on stress reduction in health workers: a controlled randomized clinical trial

**DOI:** 10.1590/1518-8345.5992.3771

**Published:** 2023-01-06

**Authors:** Kairo Silvestre Meneses Damasceno, Gerfson Moreira Oliveira, Mônica Beltrame, Julita Maria Freitas Coelho, Rodrigo Fernandes Weyll Pimentel, Magno Conceição das Merces

**Affiliations:** 1 Universidade do Estado da Bahia, Departamento de Ciências da Vida, Salvador, BA, Brazil.; 2 Escola Bahiana de Medicina e Saúde Pública, Medicina, Salvador, BA, Brazil.

**Keywords:** Auriculotherapy, Integrative Practices, Occupational Stress, Occupational Health, Epidemiology, COVID-19, Auriculoterapia, Práticas Integrativas, Estresse Ocupacional, Saúde do Trabalhador, Epidemiologia, COVID-19, Auriculoterapia, Prácticas Integradoras, Estrés Laboral, Salud Laboral, Epidemiología, COVID-19

## Abstract

**Objective:**

to assess the effectiveness of auriculotherapy in reducing occupational stress among Family Health Strategy workers during the COVID-19 pandemic.

**Method:**

a controlled randomized clinical trial divided into two groups, namely: auriculotherapy for stress group and placebo group. The Shapiro-Wilk test was used to assess data normality. The ANOVA test for repeated measures and the Tukey *post-hoc* test were applied to the group with normal samples. In turn, the Friedman and Durbin-Conover tests were employed in the group with non-normal distribution. Cohen’s d index was calculated for the therapy effect size. A 95% significance level and p<0.05 were considered.

**Results:**

the auriculotherapy group presented 16.3% and 23.7% reductions in occupational stress after the third and sixth auriculotherapy sessions, with Cohen’s d indices of 1.12 (large effect) and 1.82 (very large effect), respectively.

**Conclusion:**

auriculotherapy proved to be effective in reducing occupational stress among Family Health Strategy workers during the COVID-19 pandemic. It is suggested that new studies are developed both during and after the pandemic in order to improve health workers’ Quality of Life. ReBEC registration: RBR - 38hjyt3.

## Resumo

**Objetivo:**

avaliar a efetividade da auriculoterapia na redução do estresse ocupacional em trabalhadores de saúde da Estratégia de Saúde da Família durante a pandemia da COVID-19.

**Método:**

ensaio clínico controlado randomizado em dois grupos: grupo auriculoterapia para o estresse e grupo placebo. Foi utilizado o teste de Shapiro-Wilk para avaliar a normalidade dos dados. O teste ANOVA de medidas repetidas e o teste *post-hoc* Tukey foram aplicados para o grupo com amostras normais. Já o teste de Friedman e de Durbin-Conover foram utilizados no grupo com distribuição não normal. Para o tamanho do efeito da terapia, foi calculado o índice d de Cohen. Considerou-se o nível de significância de 95% e valor p<0,05.

**Resultados:**

o grupo auriculoterapia apresentou redução do estresse ocupacional de 16,3 e 23,7% após a terceira e sexta sessões de auriculoterapia, com índices d de Cohen de 1,12 (grande efeito) e 1,82 (efeito muito grande), respectivamente.

**Conclusão:**

a auriculoterapia mostrou-se efetiva na redução do estresse ocupacional em trabalhadores de saúde da Estratégia Saúde da Família durante a pandemia da COVID-19. Sugere-se que novos estudos sejam desenvolvidos durante e após a pandemia de maneira a melhorar a qualidade de vida dos trabalhadores de saúde. Registro ReBEC: RBR - 38hjyt3.

## Resumen

**Objetivo:**

evaluar la efectividad de la auriculoterapia para la reducción del estrés laboral en trabajadores de salud de la Estrategia Salud de la Familia durante la pandemia de COVID-19.

**Método:**

ensayo clínico controlado aleatorizado en dos grupos: grupo auriculoterapia para el estrés y grupo placebo. Se utilizó la prueba de Shapiro-Wilk para evaluar la normalidad de los datos. Al grupo con muestras normales se les aplicó la prueba ANOVA de medidas repetidas y la prueba *post-hoc* de Tukey. Se utilizaron las pruebas de Friedman y Durbin-Conover en el grupo con distribución no normal. Para el tamaño del efecto de la terapia se calculó el índice d de Cohen. Se consideró un nivel de significación del 95% y un valor de p <0,05.

**Resultados:**

el grupo auriculoterapia mostró una reducción del estrés laboral de 16,3 y 23,7% después de la tercera y sexta sesión de auriculoterapia, con índices d de Cohen de 1,12 (efecto grande) y 1,82 (efecto muy grande), respectivamente.

**Conclusión:**

la auriculoterapia demostró ser eficaz para la reducción del estrés laboral en trabajadores de la salud de la Estrategia Salud de la Familia durante la pandemia de COVID-19. Se sugiere desarrollar nuevos estudios durante y después de la pandemia con el fin de mejorar la calidad de vida de los trabajadores de la salud. Registro ReBEC: RBR - 38hjyt3.

**Highlights**

(1) Auriculotherapy is effective in reducing work-related stress among health workers.

(2) The study included Family Health Strategy professionals.

(3) Auriculotherapy proved to be a support tool during the pandemic.

(4) The auriculotherapy group presented better results than the placebo group.

## Introduction

Occupational stress is defined as chronic wear out resulting from environmental and organizational factors at work that can lead to physical, mental and behavioral changes such as high blood pressure, heart attack, anxiety, irritability, headache, physical exhaustion, gastric ulcers and insomnia, among other pathologies and symptoms^1^ ^-^ ^2^.

Health workers are exposed to a series of challenges in the work environment that are favorable to the development of occupational stress, such as interprofessional relationships, dealing with suffering, pain and even death of patients, lack of supplies, materials and equipment, high demand and working hours, situations of violence against workers, low wages and precarious working conditions^3^ ^-^ ^4^.

The COVID-19 pandemic caused by the new SARS-CoV-2 coronavirus not only intensified workload and demand, making them exhausting, but also brought about new challenges, such as fear of infection by the virus, lack of knowledge about the new disease, distance from the family and stigma towards health professionals, contributing to increased psychosocial and mental distress^5^ ^-^ ^8^.

In this sense, it is essential that actions and strategies be targeted at health workers in order to alleviate occupational stress and provide better Quality of Life. Integrative and Complementary Health Practices (ICHPs) can be an excellent alternative for the worker’s lines of care, as they are low-cost, have no side effects, and contribute to the reduction of medicalization and of the frequency of mental disorders^9^.

In the Brazilian context, in 2006, the National Policy on Integrative and Complementary Health Practices (*Política Nacional de Práticas Integrativas e Complementares em Saúde*, PNPICS) institutionalized integrative practices within the Unified Health System (*Sistema Único de Saúde*, SUS), with emphasis on Primary Care and stimulating alternatives for comprehensive and humanized care. Currently, the policy legitimizes 29 practices to be applied in the Health Care Network^10^.

Among these practices, auriculotherapy is a therapy from the Traditional Chinese Medicine that is recognized by the World Health Organization (WHO). It consists in stimulating ear points using needles, metallic spheres or seeds, to produce nerve impulses that reach the brain, stimulating the central and vegetative nervous systems, with release of endorphins, neurotransmitters and neuromediators that regulate body balance and treatment of diseases and behavioral and psychosocial disorders, such as stress^11^ ^-^ ^12^. In this direction, a number of studies already point to the effectiveness of auriculotherapy in reducing occupational stress among health workers^13^ ^-^ ^14^.

Given the above, the current study intends to assess the effectiveness of auriculotherapy in reducing occupational stress among Family Health Strategy workers during the COVID-19 pandemic.

## Method

### Study design

A single-blind controlled randomized clinical trial based on the *Consolidated Standards of Reporting Trials* (CONSORT) statement^15^.

Controlled randomized clinical trials are considered the reference standard regarding research methods in epidemiology due to better determination of an intervention efficacy. They are characterized for being experimental, having a prospective architecture, including a control group and sample randomization^16^. This randomization has the purpose of assembling groups with similar characteristics where factors that may confuse interpretation of the results have their effects neutralized from the equal distribution of these characteristics among the groups^17^.

### Locus, period and population

The research was developed with Family Health Strategy health workers (Nurses, Nursing Technicians, Dentists, Oral Health Assistants, Community Health Agents, and Physicians) from three Family Health Units (FHUs) of the Brotas Health District, municipality of Salvador, Bahia, Brazil.

The study consisted in two stages. The first stage, carried out from March to May 2021, corresponded to the epidemiological screening, where the participants signed the Free and Informed Consent Form (FICF) and answered a sociodemographic and work questionnaire and the Work-Related Stress Scale (WRSS). The inclusion criteria for the screening stage were working in the Family Health Strategy and wishing to participate in the research. The subjects excluded were those who were on leave/vacation and those who were working in the remote modality (comorbidities or pregnant women). The epidemiological screening identified 145 eligible health workers; however, 105 of them took part in this firs moment of the research.

The second stage corresponded to randomization and to the intervention, and was conducted from June to August 2021. The workers identified as having high stress levels were selected (49 participants in total), according to WRSS and, during the research, those on vacation (4) or sick leave (5), withdrawals (4) or who initiated auriculotherapy treatment by other means (3) were excluded.

### Instruments

A sociodemographic and work-related questionnaire prepared by the researchers and the WRSS scale were used.

WRSS is an instrument validated in 2004, with a Cronbach’s alpha coefficient of 0.91, that is, excellent reliability, and consists of 23 statements that associate organizational work stressors with emotional reactions. The participants answered the questions by choosing one out of five options: 1- I totally disagree; 2- I disagree; 3- I partially agree; 4- I agree; and 5- I totally agree. The highest score is assigned to the highest stress levels^18^.

Normality regarding the stress values in the sample was determined through the Kolmogorov-Smirnov test, in the screening phase. The overall mean of all the participants (2.81) was used as the cutoff point for categorizing the sample into low stress level (below the overall mean) and high stress level (above the overall mean), this being the inclusion criteria for participation in the clinical trial.

### Intervention

The intervention stage was carried out by a total of six applicators previously calibrated as to the location of the ear points and trained in auriculotherapy, where each participant was accompanied by the same therapy applicator throughout this stage.

**Figure 1:**
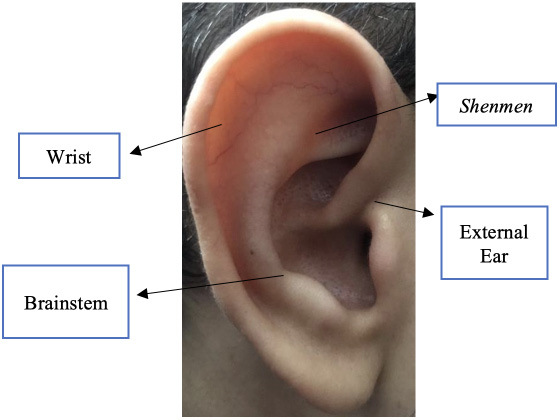
Ear points used in the auriculotherapy and placebo groups

Source: Authors’ photographic collection

Those identified with a high level of occupational stress took part in the clinical trial, totaling 49 workers (46.67% of the sample). Stratified randomization was performed in Microsoft Excel, with creation of two groups: Auriculotherapy Group (25 participants) with application at points indicated for stress (*Shenmen* and Brainstem points) and Placebo Group (24 participants) with application at points not indicated for stress (External Ear and Wrist points), as shown in Figure 1.

Stratification was performed by subdividing the high stress level values (49 participants) into higher and lower values. After the stratification process, randomization was performed so that each group (auriculotherapy and placebo) presented a more homogeneous stress distribution.

All the participants were blinded, where only the researchers aware of the therapeutic techniques knew about allocation of the volunteers to the groups.

**Figure 2:**
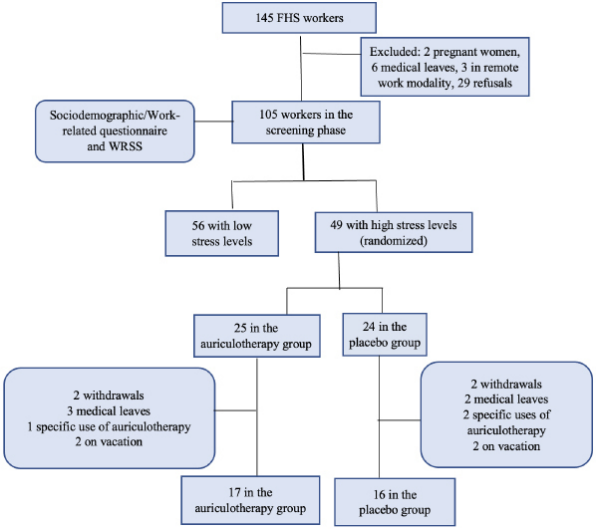
Flowchart corresponding to the research participants. Salvador, BA, Brazil, 2021

Auriculotherapy was applied 01 time *per* week for a total of six weeks, using mustard seeds fixed with hypoallergenic micropore adhesive tape. In addition to the screening phase (WRSS1), the Work-related Stress Scale was applied in the third (WRSS2) and sixth (WRSS3) auriculotherapy sessions, and 15 days after the end of the therapy (WRSS4) as a way to evaluate its residual effect. At the end of the intervention there were 17 participants in the auriculotherapy group and 16 in the placebo group. The number of participants by group is represented in Figure 2.

### Data analysis

The data collected were tabulated in Microsoft Excel. The central tendency measures were analyzed in Stata 11.0.

The Jamovi 1.6.23 program was used in the statistical analysis of the second research stage, with application of the Shapiro-Wilk test in the auriculotherapy and placebo groups to evaluate normality distribution. The ANOVA test for repeated measures was used to assess the significance of stress reduction due to the therapy and the Tukey *post-hoc* test was employed to identify which paired groups were statistically significant in cases of normal distribution. In case of non-normal distribution, the Friedman or Durbin-Conover tests were used.

Mann-Whitney’s U test was used to compare the means obtained in the auriculotherapy and placebo groups.

Cohen’s d index was also calculated to assess the therapy effect size, both in the auriculotherapy group and in the placebo group. Cohen’s d evaluates the size effect of an intervention. Values below 0.19, between 0.20 and 0,49, between 0.50 and 0,79, between 0.80 and 1.29, and above 1.30 are classified as insignificant, small, average, large and very large effect, respectively^19^.

### Ethical aspects

The research was approved by the Ethics and Research Committee (*Comitê de Ética e Pesquisa*, CEP) of the Bahia State University (*Universidade do Estado da Bahia*, UNEB) under opinion No. 4,478,349, authorized by the Personnel Training and Development Subcoordination Office of the Municipal Health Secretariat (*Secretaria Municipal de Saúde*, SMS) of Salvador under opinion No. 43/2020, and approved by the Brazilian Clinical Trials Registry (*Registro Brasileiro de Ensaios Clínicos*, ReBEC) with registration code RBR - 38hjyt3. The guidelines referring to research studies with human beings set forth in Resolution No. 466/2012 of the National Health Council were respected, as well as the principles of the Declaration of Helsinki.

## Results

There was a total of 145 Family Health Strategy (FHS) workers from all three Family Health Units. Of them, 02 were distanced due to pregnancy, 03 were working in the remote modality, 06 were on medical leave, and 29 refused to participate in the research. Thus, 11 workers were excluded from the research according to the eligibility criteria from the first stage, totaling 105 participants at the end.

The overall stress mean in the sample was 2.81, representing the cutoff point for categorizing it into low and high occupational stress level. A total of 56 participants (53.33%) presented low stress level and 49 (46.67%) were classified as with high stress level.

The absolute and relative frequencies of the sociodemographic and work-related variables in each randomized group are described in Table 1. No medical professionals took part in the intervention phase. Nurses, nursing technicians, dentists and oral health assistants were categorized as health professionals.

**Table 1:** Absolute (N*) and relative (%) frequencies of the sociodemographic variables in the auriculotherapy and placebo groups. Salvador, BA, Brazil, 2021

Variables	Auriculotherapy		Placebo	
	N*	%	N*	%
**Gender**				
Male	0	0	2	12.50
Female	17	100	14	87.5
**Race/Skin color**				
White	2	11.76	1	6.25
Black/Brown	15	88.24	15	93.75
**Professional category**				
Health Professional	6	35.28	9	56.25
Community Health Agent	11	64.72	7	43.75
**Age**				
≤ 45 years old	11	64.71	12	75.00
>45 years old	6	35.29	4	25.00
**Time working in the FHU^†^**				
≤ 7 years	7	41.18	10	62.50
>7 years	10	58.82	6	37.50
**Marital status**				
With a partner	10	58.82	7	43.75
Without a partner	7	41.18	9	56.25
**Economic situation**				
Satisfied	3	17.65	5	31.25
Dissatisfied	14	82.35	11	68.75
**Children**				
Without children	3	17.65	4	25.00
With children	14	82.35	12	75.00

*N = Absolute frequency of the participants; ^†^FHU = Family Health Units

The occupational stress mean scores in all four stages when the Work-Related Stress Scale was applied in the auriculotherapy group were as follows: 3.41 in the initial stage (WRSS1), followed by 2.86 (WRSS2), 2.60 (WRSS3) and 2.71 (WRSS4) in the subsequent stages. In the placebo group, the values found were as follows: 3.51 (WRSS1); 3.22 (WRSS2); 3.10 (WRSS3); and 3.04 (WRSS4). The mean values and standard deviations of each stage are represented in Table 2.

**Table 2:** Stress mean values and standard deviations (SD) as evaluated in all four stages (Work-Related Stress Scale - WRSS) applied in the auriculotherapy and placebo groups. Salvador, BA, Brazil, 2021

Group	Number of participants	WRSS1* - Mean (SD^||^)	WRSS2^†^ Mean (SD^||^)	WRSS3^‡^ - Mean (SD^||^)	WRSS4^§ -^ Mean (SD^||^)
Auriculotherapy	17	3.41 (±0.41)	2.86 (±0.57)	2.60 (±0.48)	2.71 (±0.36)
Placebo	16	3.51 (±0.58)	3.22 (±0.72)	3.10 (±0.73)	3.04 (±0.68)

*WRSS1 = Stress mean value in the screening phase; ^†^WRSS2 = Stress mean value after three auriculotherapy sessions; ^‡^WRSS3 = Stress mean value after six auriculotherapy sessions; ^§^WRSS4 = Stress mean value 15 days after the end of the therapy; ^||^SD = Standard Deviation

In the auriculotherapy group, the reduction of occupational stress among the participants between the first and second stages (WRSS1/WRSS2) was 16.13%, with a Cohen’s d index of 1.12 (large effect)^19^. Between the first and third stages (WRSS1/WRSS3), the reduction was 23.7%, with a Cohen’s d index of 1.82 (very large effect)^19^ and, between the first and fourth stages (which assessed the residual effect of the therapy), the stress reduction corresponded to 20.5%, with the Cohen’s d index maintaining a very large effect (1.81)^19^. The WRSS2 paired group did not present normality as per the Shapiro-Wilk test (p=0.02). The Friedman test was used in this case, which presented statistical significance (p<0.001). The Durbin-Conover test identified in which groups there was statistical significance: WRSS1/WRSS2 (p<0.001), WRSS1/WRSS3 (p<0.001) and WRSS1/WRSS4 (p<0.001).

In turn, in the placebo group the stress reduction between the first and second stages was 8.26%, with a Cohen’s d index of 0.44 (small effect)^19^. Between the first and third stages, the reduction corresponded to 11.7% and Cohen’s d was 0.62 (average effect)^19^. In turn, between the first and fourth stages, the reduction was 13.4% and Cohen’s d was 0.75, also considered as average effect^19^. As there was normality of the groups paired in the placebo group, the ANOVA analysis for repeated measures was used, finding a statistically significant relationship (p=0.01), but only between the WRSS1/WRSS4 paired groups (p=0.039). The intervention size effect through Cohen’s d index in both groups is represented in Table 3.

**Table 3:** Cohen’s d index and occupational stress percentage reduction (%) in the auriculotherapy and placebo groups. Salvador, BA, Brazil, 2021

Group	WRSS1*/WRSS2^†^		WRSS1*/WRSS3^‡^		WRSS1*/WRSS4^§^	
	Cohen’s d	%	Cohen’s d	%	Cohen’s d	%
Auriculotherapy	1.12	16.13	1.82	23.7	1.81	20.5
Placebo	0.44	8.26	0.62	11.7	0.75	13.4

*WRSS1 = Stress mean value in the screening phase; ^†^WRSS2 = Stress mean value after three auriculotherapy sessions; ^‡^WRSS3 = Stress mean value after six auriculotherapy sessions; ^§^WRSS4 = Stress mean value 15 days after the end of the therapy

The comparison between the auriculotherapy and placebo groups regarding stress at WRSS1 and WRSS4 did not present statistical significance as per Mann-Whitney’s U test, assuming p-values of 0.9 and 0.176, respectively.

No harms or undesirable effects were verified in the research participants.

## Discussion

The COVID-19 pandemic is known to have caused an increase in mental and psychosocial disorders among health workers worldwide^5^ ^-^ ^8^. The prevalence of high occupational stress levels among the Family Health Strategy professionals included in the research was 46.67%. This may have also occurred in the group herein analyzed, and contributed to the significant rate of high occupational stress levels detected.

Studies conducted with health workers in several countries around the world presented high frequencies of common mental disorders (anxiety and depression) and occupational stress during the COVID-19 pandemic. The following stand out among them: a study conducted with Nepalese individuals pointed out that 12% of the participants presented occupational stress, 30% anxiety and 22.5% depression^20^. Among Indian health workers, the values for acute occupational stress, depression and anxiety were 9.5%, 17% and 19.5%, respectively^21^. Occupational stress in health workers from a municipality in the state of Maranhão, Brazil, through the Perceived Stress Scale, presented 20.5% frequency in the very high level and 23% in the high level^22^. Among hospital health workers in Egypt, perceived stress at moderate and severe levels accounted for 98.5% of the sample, while moderate anxiety had a frequency of 32% and severe anxiety, of 18.5%^23^. In turn, in the pandemic front-line health teams from Greece, the prevalence of moderate/severe symptoms of depression, anxiety and post-traumatic stress was 30%, 25% and 33%, respectively^24^.

Faced with this context, strategies for coping with occupational stress are beneficial. Thus, ICHPs represent an important strategy for lines of care and self-care for health workers, with diverse scientific evidence for improving quality of life and reducing mental health harms during the pandemic. A study systematized experiences based on qualified listening, massage and auriculotherapy in Primary Care health workers from the city of Recife, Brazil, with appreciation of spaces for self-care and overcoming the marks left by the pandemic. The results showed good acceptance by the workers, improvements in individual and collective well-being, and pain, anxiety and stress relief, improving performance in work activities^25^.

ICHPs contribute to a reduction in mental and emotional symptoms and improve sleep quality and well-being. Therefore, they must be made available to health professionals, as well as they should be empowered both for self-care and support to others^26^.

Auriculotherapy is on the list of ICHPs and has been an important tool used for reducing psychosocial and mental distress, as well as behavioral, emotional and physiological changes. In addition to that, it is a viable practice because it is cost-effective, easy to apply and learn, fast, safe and has presented good acceptance^11^ ^,^ ^27^.

The auricular pavilion is made up of specific points whose stimulation is related to effects regarding relief of muscular and skeletal pain, treatment of mental, emotional and behavioral disorders and control of cardiovascular and gastric diseases, among others^11^ ^-^ ^12^.

The *Shenmen* point, for example, is considered a calming, analgesic, anti-inflammatory and soothing point while the Brainstem point has sedative properties and helps calm the mind. They are both indicated for stress control. The External Ear and Wrist points are indicated for problems in their respective regions; therefore, unrelated to stress^13^ ^-^ ^14^.

Applicability of auriculotherapy during the COVID-19 pandemic has been the object of some studies with satisfactory results. In an intervention research study conducted with nurses working in front line against the pandemic, a significant reduction in the participants’ mean stress scores was observed (19.37±10.61 during the pre-intervention and 11.95±8.51 in the post-intervention, p<0.001), in addition to a reduction in the depression and anxiety levels, showing the positive effect of the therapy^11^.

Research on randomized clinical trials to prove the effectiveness of auriculotherapy in reducing occupational stress is incipient, and application of this study design and theme during the COVID-19 pandemic is unknown to the authors. This hinders comparisons between studies developed in the same time context. On the other hand, this fact differentiates this study as one of the pioneers with positive results on the effectiveness of auriculotherapy in reducing stress in health professionals during the pandemic period.

In a randomized clinical trial, before the pandemic period and carried out with Nursing professionals working in hospitals, a 30% reduction in occupational stress in the auriculotherapy group (*Shenmen* and Brainstem points) was observed after 8 sessions (Cohen’s d index: 1.15), as well as a 43% decrease after 12 sessions (Cohen’s d index: 1.81), maintaining its residual effect in the follow-up period. The placebo group (Face Area and External Ear points) presented stress reductions of 17% (Cohen’s d: 0.54), 26% (Cohen’s d: 0.86) and 22% (Cohen’s d: 0.67) after 8 and 12 sessions and 15 days after the end of the therapy, respectively^14^.

Our findings presented positive results after fewer auriculotherapy sessions (6), with a higher reduction in occupational stress in the auriculotherapy group between WRSS1 and WRSS3 (23.7%), with a statistically significant difference (p<0.001) and a Cohen’s d effect of 1.82, considered very large. The placebo group also presented a 13.4% reduction in stress between WRSS1 and WRSS4, the only statistically significant relationship in this group (p=0.039), which shows the positive effects of auriculotherapy when applied to FHS workers during the COVID-19 pandemic period, when work-related stress is intensified.

Despite a lower reduction in occupational stress when compared to the auriculotherapy group, the effect in the placebo group can be due to the periodic and scheduled meetings with the participants focused on mental health care, providing a sense of support, care and welcoming through auriculotherapy, especially during the pandemic period, as cited in the literature^14^.

Most of the studies involving the auriculotherapy practice are directed at Nursing professionals and at specialized health care, to the detriment of including other health and Primary Health Care professionals. Therefore, the importance of this research is emphasized concerning the participation of health workers from the Family Health Strategy, including Nursing professionals, Dentists, Oral Health Assistants and Community Health Agents. In the pandemic context, a critical analysis of the literature showed the scarcity of studies addressing the heterogeneity of the categories of workers in coping with the health crisis, focusing on Medical and Nursing professionals at the hospital level, to the detriment of the absence of discussions about Primary Care, the population’s gateway to the Unified Health System^28^.

The method used in the research achieved positive results in reducing work-related stress among FHS workers during the COVID-19 pandemic. However, some study limitations can be pointed out, namely: the number of participants in the clinical trial (49 allocated to two groups at the beginning of the intervention) and the follow-up loss during the research (ending with 33 workers), a known disadvantage of controlled clinical trials. Lack of funding also translates as one of these limitations, as greater investments in this research might reach a much larger number of Primary Health Care professionals.

Despite these limitations, the effectiveness of auriculotherapy shown by the study evidences its relevance as a strategy for coping with stress in the health workers’ workplaces. Therefore, this line of research contributes to encouraging auriculotherapy and other ICHPs in the health services, in order to strengthen integrated and holistic health care, in accordance with the Brazilian National Policy on Integrative and Complementary Health Practices.

## Conclusion

Auriculotherapy proved to be an effective social technology in reducing occupational stress among FHS health workers during the COVID-19 pandemic with an effect considered large after 3 sessions (Cohen’s d index of 1.12 and 16.13% reduction) and a very large effect after 6 sessions (Cohen’s d index of 1.82 and 23.7% reduction), maintaining the very large effect 15 days after the end of the therapy (residual effect).

It is suggested that auriculotherapy be the object of other studies to be developed with Primary Health Care workers during or after the COVID-19 pandemic, in order to contribute to the state-of-the-art and to workers’ health and quality of life.
